# Gene expression and phytohormone levels in the asymptomatic and symptomatic phases of infection in potato tubers inoculated with *Dickeya solani*

**DOI:** 10.1371/journal.pone.0273481

**Published:** 2022-08-29

**Authors:** Iman Hadizadeh, Bahram Peivastegan, Jinhui Wang, Nina Sipari, Kåre Lehmann Nielsen, Minna Pirhonen

**Affiliations:** 1 Department of Agricultural Sciences, University of Helsinki, Helsinki, Finland; 2 College of Plant Protection, Hebei Agricultural University, Hebei, China; 3 Viikki Metabolomics Unit, Faculty of Biological and Environmental sciences, University of Helsinki, Helsinki, Finland; 4 Department of Chemistry and Bioscience, Aalborg University, Aalborg, Denmark; Hainan University, CHINA

## Abstract

*Dickeya solani* is a soft rot bacterium with high virulence. In potato, *D*. *solani*, like the other potato-infecting soft rot bacteria, causes rotting and wilting of the stems and rotting of tubers in the field and in storage. Latent, asymptomatic infections of potato tubers are common in harvested tubers, and if the storage conditions are not optimal, the latent infection turns into active rotting. We characterized potato gene expression in artificially inoculated tubers in nonsymptomatic, early infections 1 and 24 hours post-inoculation (hpi) and compared the results to the response in symptomatic tuber tissue 1 week (168 hpi) later with RNA-Seq. In the beginning of the infection, potato tubers expressed genes involved in the detection of the bacterium through pathogen-associated molecular patterns (PAMPs), which induced genes involved in PAMPs-triggered immunity, resistance, production of pathogenesis-related proteins, ROS, secondary metabolites and salicylic acid (SA) and jasmonic acid (JA) biosynthesis and signaling genes. In the symptomatic tuber tissue one week later, the PAMPs-triggered gene expression was downregulated, whereas primary metabolism was affected, most likely leading to free sugars fueling plant defense but possibly also aiding the growth of the pathogen. In the symptomatic tubers, pectic enzymes and cell wall-based defenses were activated. Measurement of hormone production revealed increased SA concentration and almost no JA in the asymptomatic tubers at the beginning of the infection and high level of JA and reduced SA in the symptomatic tubers one week later. These findings suggest that potato tubers rely on different defense strategies in the different phases of *D*. *solani* infection even when the infection takes place in fully susceptible plants incubated in conditions leading to rotting. These results support the idea that *D*. *solani* is a biotroph rather than a true necrotroph.

## Introduction

Soft rot bacteria are devastating plant pathogens that collectively cause rotting in hundreds of plant species [1]. They belong to the *Pectobacterium* and *Dickeya* genera in the *Pectobacteriaceae* family. Currently, 19 *Pectobacterium* species and 12 *Dickeya* species have been suggested or formally accepted on the List of Prokaryotic names with Standing Nomenclature (LPSN.dsmz.de). *D*. *solani* isolates were identified in several countries in Europe in the early 2000s as highly virulent soft rot pathogens and were later verified as a new species linked to increased losses in potato cultivation in Europe [2]. *D*. *solani* has limited genetic diversity and causes diseases in hyacinth and potato. Hyacinth has been suggested as the original host from which the bacteria moved to potato in the Netherlands [3]. Due to the relatively recent identification of *D*. *solani*, knowledge about its virulence mechanisms and plant response to *D*. *solani* need to be interpreted from results generated using *Pectobacterium* and other *Dickeya* species [4].

Soft rot bacteria are ubiquitous in nature and are residents of various environmental niches from weeds to natural waters and insects [5]. In potato, *D*. *solani*, like the other potato-infecting soft rot bacteria, causes rotting and wilting of the stems and rotting of tubers in the field and in storage. Soft rot bacteria can survive in the plants for several plant generations as latent infections without causing symptoms, but various environmental and physiological conditions, such as high humidity, hypoxic conditions, reduced dormancy and tuber senescence during storage, can reduce the resistance of the tubers and allow the bacteria to grow to high numbers [5, 6]. Latent, asymptomatic infections of soft rot bacteria are common in potato, which makes the control of soft rot diseases very challenging and often leads to rotting of the tubers during storage [7].

Soft rot bacteria synthesize and secrete plant cell wall-degrading enzymes (PCWDEs) in plant intercellular spaces to macerate host tissues and obtain nutrients from the dead cells [8, 9]. PCWDEs are involved in virulence but also alert the plant to the presence of the pathogen because pectic fragments produced by PCWDEs are recognized by plants as danger-associated molecular patterns (DAMPs) [10]. The production of PCWDEs by *Pectobacterium* activates genes associated with JA biosynthesis, ET response, oxylipin biosynthesis and response to wounding but also innate immunity and cell death [11]. Furthermore, soft rot bacteria produce necrosis-inducing toxins [12] and contain structures, such as flagella, elongation factor Tu, lipopolysaccharides and peptidoglycans, that are detected by the plant as pathogen-associated molecular patterns (PAMPs) that induce PAMPs-triggered immunity (PTI) in infected plants [13]. Most *Pectobacteriaceae* species harbor a Type III secretion system (T3SS) and produce HrpN and other helper proteins, but only one effector, the AvrE-family effector DspE, that causes cell death in leaf tissue at the beginning of the infection [14]. DspE is needed for full virulence of the pathogen in tuber tissue [15], but it does not suppress innate immunity in the host [16]. In the interactions between biotrophic pathogens and resistant plants, T3SS effectors lead to effector-triggered immunity (ETI) that enhances the plant defenses and causes a hypersensitive response (HR), a form of programmed cell death [17]. In the interactions between soft rot bacteria and their hosts, the roles of HR and programmed cell death are not clear, as the death of the plant cells can be speculated to benefit the pathogen [18].

Recognition of DAMPs, PAMPs and effectors during PTI and ETI leads to the production of reactive oxygen species (ROS), activated Ca^2+^-mediated responses, stimulation of mitogen-activated protein kinase (MAPK) cascades and production of plant hormones [19]. Different plant hormones may act downstream of PTI or ETI in the interaction to protect the plants against biotrophs and necrotrophs: SA protects the plants against biotrophic and hemibiotrophic pathogens, whereas JA and ET have roles in the defense against necrotrophic pathogens [20]. Soft rot bacteria have been considered typical necrotrophs that kill their host, but recently, they have been identified as hemibiotrophs due to their ability to exist in plant tissue as latent infections and because they can upregulate genes involved in PTI- and SA-mediated defenses, especially during latent infection [9, 21].

Due to the importance of both asymptomatic and symptomatic infections in the epidemiology of *D*. *solani* on susceptible potato tubers, we employed time-course RNA-Seq analyses at early stages at 1 and 24 hours post-inoculation (hpi) and compared them to results obtained from symptomatic tubers one week (168 hpi) later. The results were verified with qRT–PCR and metabolomics analysis of the defense-related hormones SA and JA. The results suggest that even in environmental conditions leading to compatible interaction, the tubers showed induction of genes involved in PTI and ETI-like responses and induced SA production at the early time points, whereas increased JA production was induced in the symptomatic tubers.

## Materials and methods

### Plant material, bacterial strain, inoculation and sampling

The potato cultivar ‘Bintje’ was used in the RNA-Seq profiling experiment, obtained from Finnish Seed Potato Centre Ltd. and stored in a cold room at 4 °C before the experiments. A Finnish highly aggressive *Dickeya solani* isolate (strain Ds0432-1) [22] was provided by the bacterial collection of the Plant Pathology Laboratory of the University of Helsinki. The bacterial strain was stored in cryovials with 30% glycerol at −80 °C. For tuber infection, one bacterial colony grown for 48 h at 28 °C on Luria-Bertani (LB) agar (Merck, Darmstadt, Germany) was transferred to LB broth (5 ml) and cultured overnight on a rotary shaker (200 rpm) at 28 °C. Overnight cultures were centrifuged at 7000 g for 10 min and harvested in sterile water, and then the bacterial solutions were adjusted to an OD_600_ of 0.2 (5 × 10^8^ CFU.ml^−1^), unless otherwise stated. The potato tubers were washed thoroughly in tap water, surface sterilized by immersion in 3% sodium hypochlorite for 5 minutes and dried for 45 min at room temperature in the dark before being used for inoculation. A hole was made on top of each tuber by piercing with a 1000-μl pipette tip, and 50 μl of bacterial suspension or water (mock treatment) was deposited into each hole. After inoculation, the holes were covered with Vaseline to preserve humidity. Inoculated tubers were placed on stainless sieves in polyethylene boxes internally lined with moist cloth to maintain medium humidity (70–80%), and the closed boxes were incubated at 15 °C in the dark, a temperature that allows bacterial growth. For RNA-Seq, inoculated tubers and controls were cut in half, and a 0.5 cm× 0.5 cm tissue sample, including cells from the periderm, cortex and medulla layers, was collected using a scalpel from the healthy-looking tissue beside the inoculation point. The samples were frozen immediately in liquid nitrogen and stored at -80 °C prior to RNA extraction. The same sampling method was used for quantitative real-time PCR (qRT–PCR) and phytohormone analyses, each in a separate experiment.

### Tuber soft rot assay and quantification of bacteria in potato tissue

A tuber soft rot assay was used to evaluate the soft rot symptoms and severity caused by *D*. *solani* inoculation. Tubers were inoculated as described above with 20 tubers in each treatment and incubated at 15 °C in the dark. Disease development was observed, and severity was measured at 24 and 168 hpi by calculating the weight of macerated tissue of each tuber before and after removal of rotten tissue, and the average weight of rotting tuber tissue (in grams) was determined. This experiment was repeated twice.

### RNA-Seq experimental design and sequencing

A time-course RNA-Seq experiment was employed to analyze the transcriptional response of potato tubers to *D*. *solani* infection at 1, 24 and 168 hpi. Total RNA was extracted directly from dissected frozen materials of each sample by the CTAB method [23]. A TURBO DNA-free kit (Ambion) and an RNeasy MinElute clean-up kit (Qiagen) were used for DNase treatment and RNA purification, respectively, according to the manufacturer’s instructions. RNA purity and quantity were assessed by Nanodrop (Thermo Scientific Nanodrop 2000), and RNA integrity was checked on a 1% agarose gel. A Ribo-zero kit was used to remove unwanted ribosomal RNA (r-RNA), and a cDNA library was constructed according to a high-throughput Tru-Seq RNA Sample Preparation protocol containing two rounds of isolation of poly-A RNA using oligo-DT magnetic beads. Sequencing was performed on an Illumina HiSeq 2000 platform. Through base calling, the original image data from the sequencing platform were transferred into the corresponding nucleotide sequence data FASTQ file. FASTQC (V0.10.1) was used to determine the quality of the RNA-Seq data (http://www.bioinformatics.babraham.ac.uk/projects/fastqc/). Prior to mapping reads to the reference database, reads were trimmed by eliminating reads with low-quality sequences, with unknown nucleotides larger than 5% and without Illumina adaptors. Thereafter, sequencing data were evaluated by assessing base composition, quality distribution and the saturation of the sequencing data. At the same time, the Q20, Q30, GC content and sequence duplication level of the clean data were calculated. All downstream analyses were based on clean, high-quality data. The draft genome of potato (PGSC_DM_v3_2.1.10, http://potatogenomics.Plant-biology.msu.edu/index.html) at http://solgenomics.net/organism/Solanum_tuberosum/genome was used for reference-guided mapping of transcriptome sequencing reads.

### Sequencing data analysis

RNA-Seq data were analyzed as described earlier [24]. Briefly, the potato genome sequence (PGSC_DM_v4.03) was used to build the mapping index using Bowtie2 (v2.1.0), and clean reads were then aligned to the indexed genome using TopHat (v2.1.1). The mapping parameters were “-p 8 —b2-very-sensitive—solexa-quals—segment-length 30—segment-mismatches 3—mate-std-dev 20—library-type fr-unstranded” (http://ccb.jhu.edu/software/tophat/index.shtml). The relative abundances and differences between treatments were calculated using Cufflinks (v2.2.1) with default settings [25]. A correlation coefficient of >0.90 was performed in each library based on the fragments per transcript kilobase per million fragments mapped (FPKM) value, and the cutoff value was the 95% confidence limit of the FPKM for all genes. Statistically significant differentially expressed genes (DEGs) were identified by NOISeq analysis with the R/Bioc package using read counts with probability > 0.75, p-value < 0.05 and log2 fold change ≥ 9 and ≤ −9 [26, 27]. To visualize gene expression, hierarchical cluster analysis was carried out by Cluster 3.0 and visualized using Java TreeView software. The parameters used in Cluster 3.0 were “-g 7 -e 7 -m a” (http://bonsai.hgc.jp/~mdehoon/software/cluster/software.htm).

To investigate the biological function and involvement in functional pathways, all the identified transcripts were mapped to the Gene Ontology (GO) and Kyoto Encyclopedia of Genes and Genomes (KEGG) databases. For GO annotation, the transcripts were subjected to BLASTX searching against the NCBI nonredundant (Nr) database with a typical cutoff *E*-value ≤ 10^−5^. Then, GO annotation and functional classifications were assigned by Blast2GO [28] and WEGO software [29]. The metabolic pathway annotation and enrichment of the DEGs were performed by KEGG (KEGG- http://www.genome.jp/kegg/) to define the main statistical metabolic pathways involved. GO and KEGG terms with a corrected p-value < 0.05 were considered significantly enriched for the DEGs.

### Real-time PCR

To validate the RNA-Seq data, qRT–PCR was carried out to determine the target gene expression levels in different treatments and time points. In the RNA-Seq data, the DMT number (such as PGSC0003DMT400039410) was used to retrieve the transcript sequence from the transcript reference file (PGSC_DM_v3.4_transcript_representative.fasta.zip) from the SOL genomic network. PrimerQuest (http://www.eu.idtdna.com/Primerquest/Home/Index) from Integrated DNA Technologies (IDT) was used to design the primers. The specificity of the primers was checked in a 1% agarose gel and monitored by the presence of a single peak in the melting curve after performing qRT–PCR. To make cDNA, clean total RNA (1 μg) was reverse transcribed using Enhanced Avian Reverse Transcriptase (Sigma, A 4464) following the manufacturer’s instructions. An epMotion^®^ 5075 pipetting robot (Eppendorf) was used for all PCR pipetting and qRT–PCR experiments performed on a LightCycler^®^ 480 Real-Time PCR System (Roche) with three replicates of independent cDNAs. The amplicon of each target gene was visualized on a 1% agarose gel to verify the amplification of a single product with the expected length. The relative fold changes in the expression levels of the target genes were normalized to eukaryotic initiation factor 5A as a housekeeping gene [30], and log2 expression was calculated by 2^–ΔΔCT^ based on the Livak method [31]. The qRT–PCR experiments were performed three times with three technical replicates of each cDNA, with similar results, and the data for each experiment were analyzed separately.

### Extraction and quantification of phytohormones

The phytohormones SA and JA were analyzed from inoculated tubers at 24 and 168 hpi as described earlier [24]. Approximately 400 mg (FW, fresh weight) of snap-frozen, ground tuber sample was extracted twice with 1 ml of an ice cold (− 20 °C) methanol: isopropanol: acetic acid (20:79:1) mixture [32]. Five microliters of internal standard mix (ISTD; SA-d4, dh-JA; 100 ng/ml) was added. The extracts were evaporated to dryness, reconstituted in 50 μl acetonitrile (ACN) and then run immediately in randomized order with an ultra-performance liquid chromatography-tandem mass spectrometer (UPLC–MS). Four replicate samples, each containing ground tissue from five tubers, were analyzed. The results of SA and JA were normalized to the corresponding deuterated ISTDs (SA-d4 and dh-JA, respectively) and FW and were quantified using calibration curves for each phytohormone with Analyst MultiQuant^™^ software (ABSciex Pte. Ltd.).

The UPLC–MS system consisted of ExionLC UPLC connected to a Sciex QTRAP-6500+ via ESI (AB Sciex Pte. Ltd.) The mobile phase consisted of 0.1% formic acid in MQ water (A) (Merck Millipore) and ACN (B) (Honeywell, Riedel-de Haën, CHROMASOLV^™^, LC–MS grade). The chromatographic separation was performed in a Waters Acquity UPLC BEH C18 (Ø 1.7 μm, 2.1 mm × 50 mm) column (Waters) with a flow rate of 0.6 ml/min and a linear gradient of 5 to 75% B over 7 min. The injection volume was 5 μl. The mass spectrometer was operated in multiple reaction mode (MRM) with polarity switching (ESI+/−) using optimized precursor-to-product ion transitions due to its high selectivity and increasing sensitivity for low-abundance phytohormones. The MRMs used for each phytohormone and their corresponding ISTDs were 136.84 > 92.9 (ESI-) for SA, 140.88 > 97.0 (ESI-) for SA-d4, 208.95 > 59.0 (ESI-) for JA and 210.97 > 58.9 (ESI-) for dh-JA.

## Results and discussion

### RNA-Seq and transcriptome profiles of potato tubers in response to *D*. *solani*

**Fig 1:**
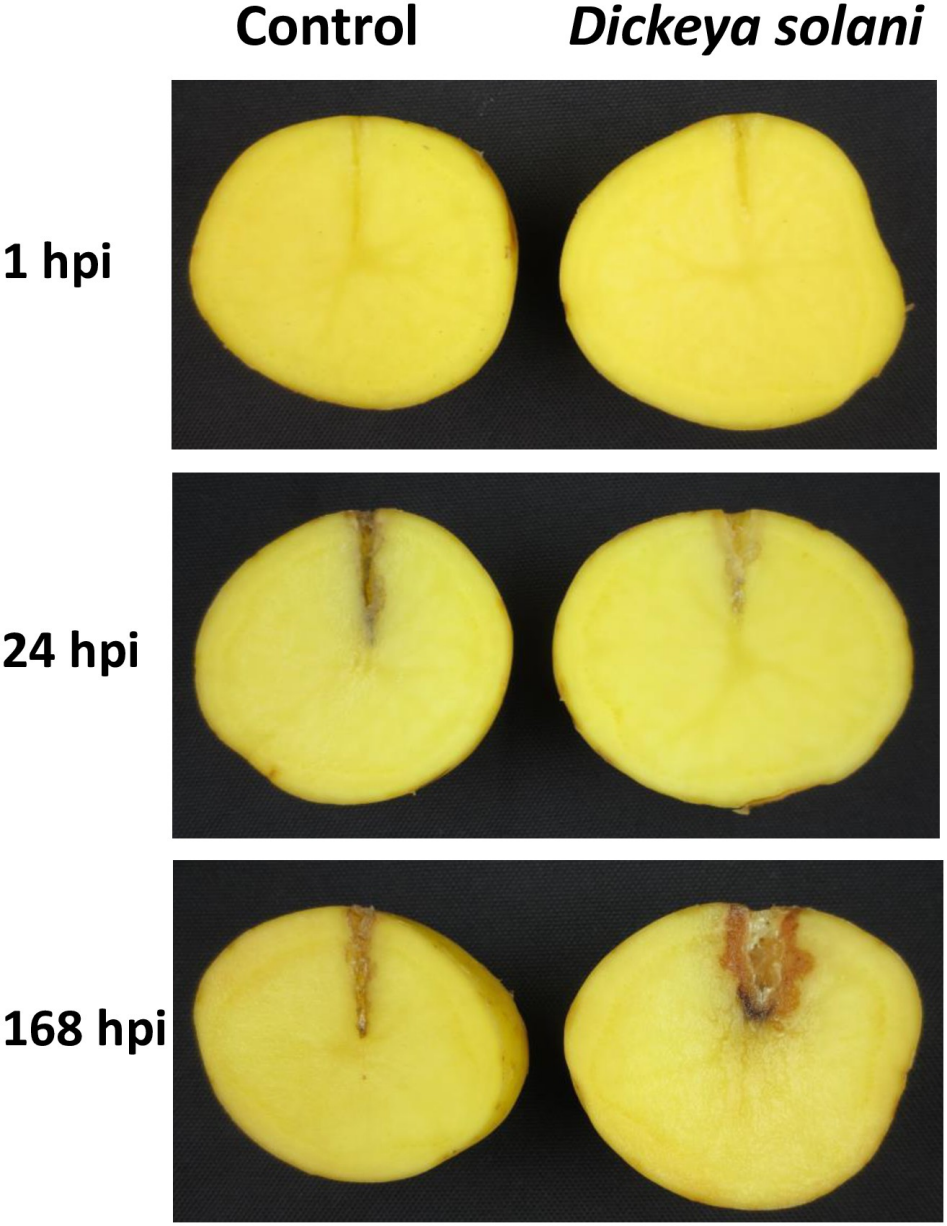
Symptoms caused by *Dickeya solani* infection in potato tubers 1, 24 and 168 hours postinoculation (hpi). Each tuber was inoculated with suspension containing 2.5 × 10^7^ CFU of *Dickeya solani* in 50 μl of sterile water. Sterile water was used as a negative control.

**Fig 2:**
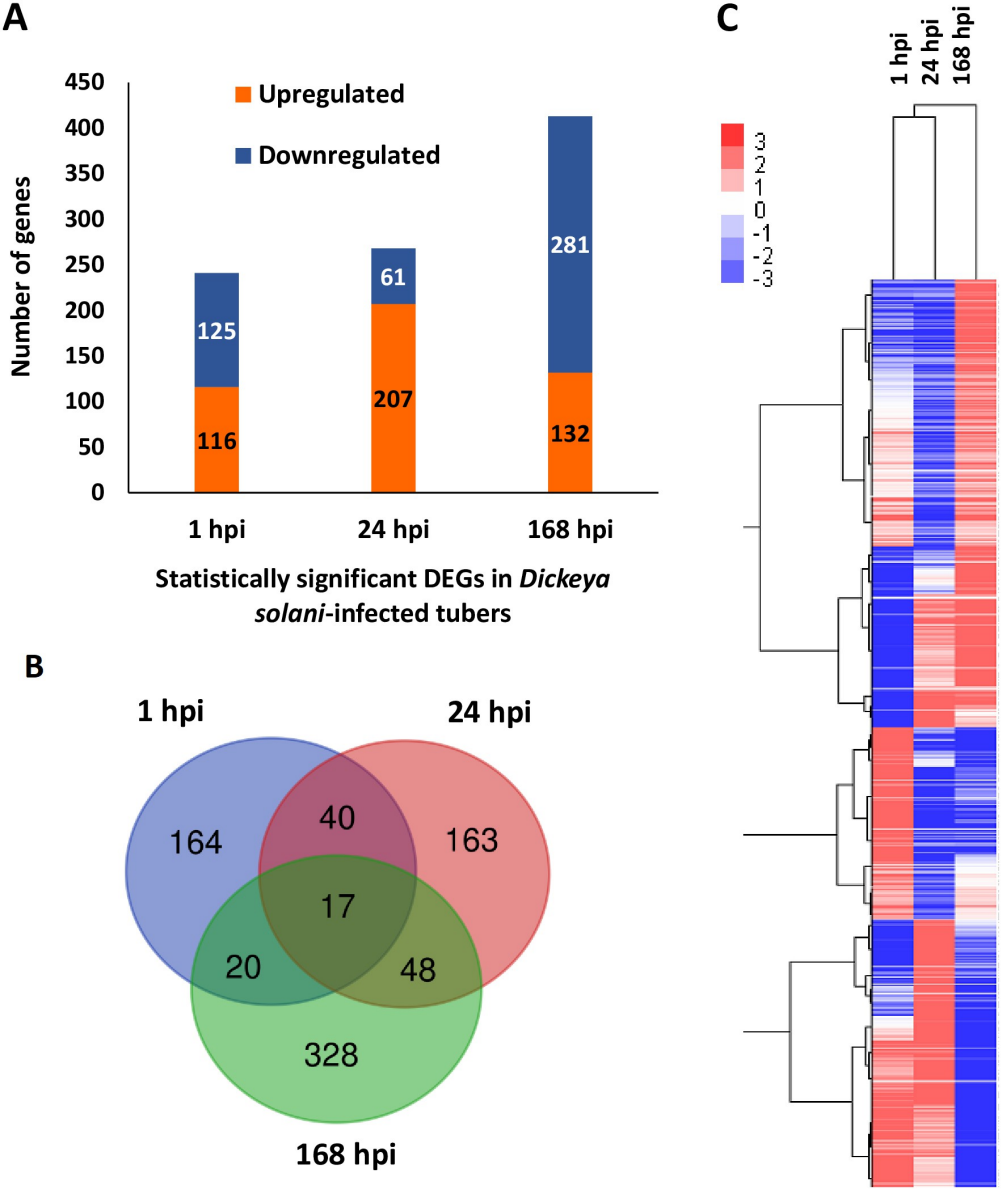
Gene expression changes in potato tubers 1, 24 and 168 h postinoculation (hpi) with *D*. *solani*. **A** The numbers of statistically significant DEGs that were either up- or downregulated at each time point (1, 24 and 168 hpi) in comparison between pathogen- and mock-treated control tubers. **B** Venn diagram analysis of the DEGs showing the overlaps of the genes between different time points in each comparison. **C** Heatmap based on clustering of 542 DEGs identified with NOISeq with probability > 0.8, p-value < 0.05 and log2 fold change ≥ 2 and ≤ −2 at one or more of the time points. Hierarchical clustering suggests that the 1 and 24 hpi samples clustered together. The scale bar represents relative expression values, red indicates upregulated genes, blue indicates downregulated genes and white indicates genes whose expression levels were unchanged between inoculated and control tubers.

The soft rot development and disease severity on potato tubers after 24 and 168 hpi inoculation with *D*. *solani* were measured by tuber soft rot assay. At 24 hpi, some tissue softening (0.54 g/tuber, SD 0.09 g) and brown tissue coloring were observed in the inoculation site, but no noticeable soft rot symptoms were observed in the inoculated tissue (Fig 1). In the late stage at 168 hpi, browning and rotting of the infected tissue were evident (3.28 g/tuber, SD 1.11 g). Some browning but no maceration symptoms were found in the mock-treated tubers. The RNA-Seq experiment was designed to analyze the transcriptional response of potato tubers to *D*. *solani* infection at the early stages when no symptoms were visible (1 and 24 hpi) and at the later stage (168 hpi) when clear rotting was obvious and water-treated mock tubers served as the controls. In total, eighteen libraries were subjected to RNA-Seq to generate approximately 140 million 100-bp paired-end reads with an average of 7.5 million reads in each individual library. The expression profiles of 14,583 genes were identified by merging the transcripts reconstructed from each sample using the Cufflinks software tool (v2.2.1) (25). An overview of the sequencing and mapping results is shown in Table 1, and the primary dataset is available in S1 Table. NOISeq analysis was performed to identify differential expression (S2 Table), and the statistically significant DEGs were determined to have probability > 0.75, p-value < 0.05 and fold change > 9 and < −9 (S3 Table). Expression profiles revealed a total of 241, 268 and 413 statistically significant DEGs in pathogen-inoculated samples compared to mock-inoculated controls at 1, 24 and 168 hpi, respectively (S3 Table, Fig 2A). To analyze the overlap between DEGs at different time points, Venn diagram and clustering analyses were carried out. The results showed that the early time points 1 and 24 hpi were closer to each other than to the late time point 168 hpi (Fig 2B and 2C).

**Table 1:** RNA-Seq data. Summary of paired-end sequenced data, mapping and reference-based assembly determined by RNA-Seq analysis in potato tubers that were inoculated with *Dickeya solani* (Ds) and control (C) at time points 1, 24 and 168 h post-inoculation (hpi).

Library Name	Reads	% of Mapped Reads	Average %
R1-1H-C	8,554,178	70.21	
R2-1H-C	7,658,423	68.44	68.05
R3-1H-C	6,931,578	65.51	
R1-1H-Ds	9,521,480	78.98	
R2-1H-DS	7,982,154	72.71	77.68
R3-1H-DS	8,089,451	81.36	
R1-24H-C	8,369,481	63.65	
R2-24H-C	7,901,547	69.47	67.66
R3-24H-C	6,089,478	69.86	
R1-24H-DS	6,879,453	71.74	
R2-24H-DS	9,598,547	65.68	69.94
R3-24H-DS	6,674,851	72.39	
R1-168-C	7,845,171	65.96	
R2-168-C	8,395,685	72.38	68.51
R3-168-C	5,298,478	67.19	
R1-168-DS	9,221,548	79.35	
R2-168-DS	7,874,152	76.44	76.36
R3-168-DS	6,395,985	73.29	
Total	139,281,640		

**Fig 3:**
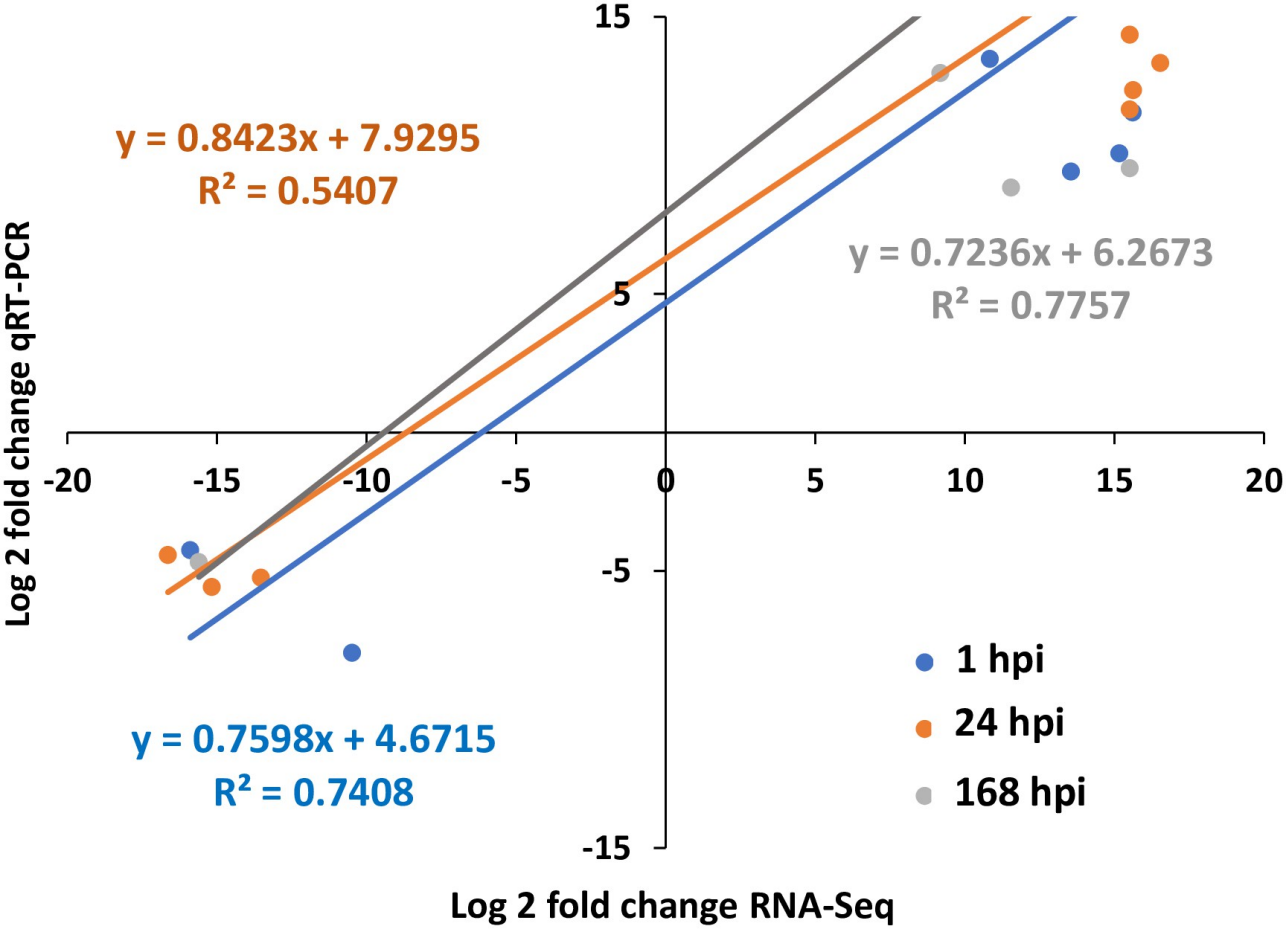
qRT–PCR validation of DEGs obtained from RNA-Seq sequencing of potato tubers inoculated with *D*. *solani*. Trend lines of qRT–PCR validation of time-course RNA-Seq, RNA-Seq data (x-axis) and qRT–PCR (y-axis) data (log2 fold change) analyzed by the Pearson test (p < 0.05). The trend line equation and the corresponding square regression coefficient (R2) are shown. The statistical analysis for the data was performed with coefficient correlation analysis between RNA-Seq and qRT–PCR data (log2 fold change) analyzed by the Pearson test (p < 0.05), which resulted in a strong correlation between the analysis methods. Bar graphs of the individual genes are shown in S1 Fig, primer sequences and full names of the genes are shown in S4 Table, and the primary qRT–PCR data are shown in S5 Table.

To validate the transcriptome profiling results, real-time qRT–PCR analysis was performed on 23 identified genes (S4 and S5 Tables; Fig 3). Although the fold changes in transcript abundance determined by RNA-Seq and qRT–PCR did not exactly match, all of the qRT–PCR analyses showed trends of expression (up- or downregulation) similar to those revealed by the RNA-Seq results. These results confirmed the accuracy of our RNA-Seq data. The correlation coefficient of the gene expression trends in the sequencing data and qRT–PCR results was 0.813 (Fig 3).

### GO and KEGG pathway enrichment

**Fig 4:**
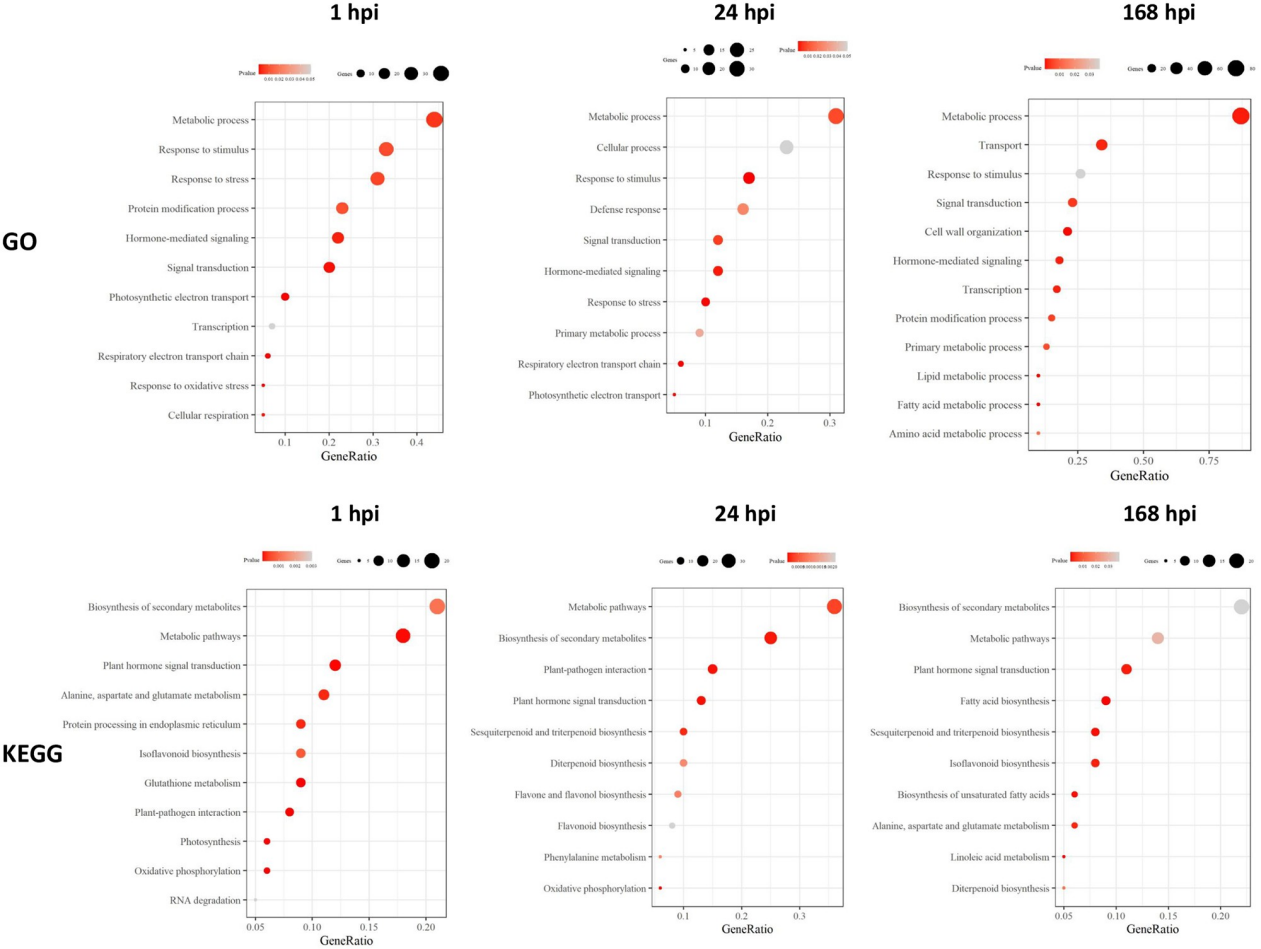
Enriched GO and KEGG terms among the statistically significant differentially expressed genes at 1, 24 and 168 h postinoculation (hpi) with *D*. *solani*. The genes were chosen based on NOISeq analysis with probability > 0.8, p-value < 0.05 and fold change > 9 and < −9.

GO enrichment analysis was performed to analyze the functions of statistically significant DEGs in response to *D*. *solani* infection over time. In total, 177 of 241 DEGs at 1 hpi, 171 of 268 DEGs at 24 hpi and 279 of 413 DEGs at 168 hpi were assigned to at least one term of the three major categories: biological process (BP), molecular function (MF) and cellular component (CC) (S3 Table). BP responses were the most informative, so we focused on them. Several BP GO terms were shared between all three time points, such as “metabolic process”, “response to stimulus”, “signal transcription”, and “hormone-mediated signaling” (Fig 4). The early time points 1 and 24 hpi shared the GO terms “response to stress”, “respiratory electron transport chain” and “photosynthetic electron transport”, and 24 and 168 hpi shared the GO term “primary metabolic process”. The most significantly enriched unique GO terms at the 24 hpi time point were “cellular process” and “defense response”, and at the late time point (168 hpi), they were “transport”, “cell wall organization”, “lipid metabolic process”, “fatty acid and lipid metabolic process” and amino acid metabolic process (Fig 4). Time points 1 and 168 hpi shared several GO terms, but a closer look at the genes showed that many of the genes were different or regulated in opposite directions (S3 Table).

The DEGs were mapped on KEGG to retrieve the significant pathways. Nearly 40% of the DEGs were mapped to pathways, whereas the rest were unassigned. All three time points shared “biosynthesis of metabolic pathways”, “metabolic pathways” and “plant hormone signal transduction”, and all time points shared enriched KEGG pathways of isoflavonoid or flavonoid biosynthesis (Fig 4). “Plant–pathogen interaction” and”oxidative phosphorylation” were shared by the early time points 1 and 24 hpi, and “protein processing in endoplasmic reticulum”, “glutathione metabolism”, “photosynthesis” and “RNA degradation” were found to be enriched only at 1 hpi, while “phenylalanine metabolism” was enriched only at 24 hpi (Fig 4). Similar to GO terms, in KEGG terms, changes in fatty acid production at 168 hpi were evident with “fatty acid biosynthesis”, “biosynthesis of unsaturated fatty acids” and “linoleic acid metabolism”, which were enriched only at 168 hpi. Furthermore, 24 and 168 hpi shared “sesquiterpenoids and triterpenoid biosynthesis” and “diterpenoid biosynthesis” (Fig 4).

### Early response of asymptomatic potato tuber to *Dickeya solani* inoculation

Due to the similarity of the 1 and 24 hpi results, they are described here jointly. Among the identified statistically significant DEGs, many upregulated genes were involved in pathogen recognition (S3 Table). Upon closer inspection of the KEGG pathway “plant–pathogen interaction” and the individual upregulated DEGs, a number of genes involved in PTI-related responses were identified. Among them were a gene identified as FLS2, although with low homology, and a gene homologous to the PTI1-like tyrosine-protein kinase At3g15890-like gene that contributes to the production of ROS in response to *Pseudomonas syringae* flagellin-derived peptides in tomato [33]. Furthermore, we identified a probable LRR receptor-like serine/threonine-protein kinase At1g07650-like similar to Arabidopsis LMK1 involved in cell death induction [34], a hypothetical protein (XLOC_018313) that is similar to the LRR receptor-like serine/threonine-protein kinase NILR2 involved in the response of Arabidopsis to LPS of *Xanthomonas campestris* [35], an LRR receptor-like serine/threonine-protein kinase RKF1 that is induced by SA in Arabidopsis [36] and a cysteine-rich receptor-like protein kinase 2-like similar to Arabidopsis RLK2/CRK2 needed for PAMPs-triggered ROS production and immunity against *Pseudomonas tomato* [37]. Mitogen-activated protein kinase kinase kinase (MAPKKK), a subset of genes related to activation of Ca^2+^ internal flow (e.g., CNGC, CML, CDKs) and respiratory burst oxidase homolog NADPH oxidase RBOHB were induced in the early samples. Furthermore, WRKY transcription factor 70 (WRKY70) and WRKY3 involved in SA-induced gene expression in other plants [38, 39] and two pathogenesis-related protein 1 (PR1) homologs were upregulated in *D*. *solani*-infected tubers at one or both of the early time points (S3 Table). In conclusion, comparison of the responses of potato to other plants revealed that potato tubers may react with the PTI response to various *D*. *solani* PAMPs during the first hours of contact.

Several DEGs encoding resistance (R) proteins were differentially expressed at both 1 and 24 hpi (S3 Table). R proteins are involved in the activation of ETI responses [40]. They contain a nucleotide-binding (NB) site and a leucine-rich repeat (LRR) domain and are divided into two broad classes based on their N-terminal structures, with either a toll/interleukin (TIR) domain or a coiled-coil (CC) domain. Both kinds of R genes were identified among the DEGs, and most of them were upregulated. Furthermore, the serine/threonine-protein kinase At5g01020-like protein, similar to Arabidopsis RPM1-INDUCED PROTEIN KINASE (RIPK) involved in the activation of innate immunity, and the pathogenesis-related gene transcriptional activator Pti5 were upregulated at 1 hpi. Pti5 activates the expression of a wide array of PR proteins and plays an important role in plant defense in tomato [41]. Furthermore, hypersensitive-induced response protein 1-like (HIR1), which promotes cell death [42], was upregulated at 24 hpi. Potato stems inoculated with *Pectobacterium brasiliense* showed upregulation of R genes; however, the upregulation was not correlated with the susceptibility of the cultivar and may thus not be directly connected to defense [43]. It is possible that PTI induction leads to the induction of R genes in potato, as crosstalk between PTI and ETI responses has been suggested in other plants [44].

Numerous PR protein genes were identified in the data in addition to PR1, especially in the 24 hpi sample. Among them were wound-induced WIN2, similar to hevein-like protein, pathogen-related protein β-1,3-glucanase (PR2), fungal cell wall degrading enzyme endochitinase (PR3) with high antifungal activity, osmotin-like protein (PR5) and pathogenesis-related protein P69G, all with upregulated expression at 24 hpi. Additionally, plasma membrane-localized syntaxins (e.g., SYP131, SYP121, SYP22), trafficking protein particle complexes and vesicle-associated membrane proteins (VAMPs), which drive immune exocytosis by forming the SNARE complex [45, 46], were upregulated at early time points. It has been suggested that the SNARE complex is essential for plant exocytosis-associated immunity, secretion of toxic molecules and penetration resistance in pathogen attack sites [46, 47]. In tobacco, syntaxin was reported to play a role in PR1 accumulation and defense against bacterial pathogens in *Nicotiana benthamiana* [48].

DEGs associated with the cell wall, including xyloglucan endotransglucosylase/hydrolase and expansins, were upregulated, while cellulose synthase was downregulated at 24 hpi, suggesting changes in cell wall architecture in wounded and infected potato tissue, possibly causing induced cell wall-associated defense [49]. Moreover, defense signaling triggered the expression of genes encoding proteinase inhibitors (PIs), annotated as potato type I (Pin1) and II (Pin2) proteinase inhibitors, Kunitz-type trypsin inhibitors, cysteine protease inhibitor 1, aspartic proteinase-like protein 2 and miraculin-like proteins (MLPs). In potato, protease inhibitors are the most abundant group of proteins that, in addition to their role in regulating proteolytic activities, display a role in plant defense against pathogens [50]. For example, MLPs are members of the plant Kunitz serine trypsin inhibitor (STI) family of PIs that are involved in plant defense against many bacterial and fungal pathogens and respond to other factors, such as wounding and insect attack [51, 52]. Both cell wall-associated defenses, wound response and induction of proteinase inhibitors are regulated by JA, which suggests that JA was produced in potato tubers at early time points.

Activation of plant immune responses is associated with rapid production of vast amounts of ROS that dramatically alter cellular redox homeostasis and activate the oxidative burst. ROS generation during the PAMPs-triggered oxidative burst is predominantly achieved by apoplastic NADPH oxidases [53]. At 1 hpi, genes encoding RBOH and Rac-like GTP-binding protein, which participate in the regulation of NADPH oxidase-dependent ROS production in elicitor signaling [54], were upregulated (S3 Table). Simultaneously, ROS scavenging systems were influenced during pathogen-inducible oxidative stress at 1 hpi and less so at 24 hpi (S3 Table). For example, most glutathione S-transferase and thioredoxin genes were significantly upregulated only at 1 hpi, while glutaredoxins and nucleoredoxin genes showed upregulation at both time points.

KEGG analysis identified the biosynthesis of secondary metabolites, especially in the 24 hpi data (Fig 4). A large number of genes involved in phenylpropanoid, flavonoid and terpenoid biosynthesis were identified among the upregulated genes. Induction of genes encoding phenylalanine ammonia-lyase, chorismate mutase, trans-cinnamate 4-monooxygenase, 4-coumarate-CoA ligase and cinnamyl alcohol dehydrogenase are in the phenylpropanoid pathway that leads to SA and lignin biosynthesis. The corresponding monolignols of lignin are derived from phenylalanine ammonia-lyase via a series of enzymatic reactions. The other genes encoding these enzymes, including cinnamoyl CoA reductase-like, shikimate O-hydroxycinnamoyltransferase, caffeoyl-CoA O-methyltransferase and cinnamyl alcohol dehydrogenase, were also upregulated upon *D*. *solani* infection at 24 hpi. Additionally, genes annotated as flavonoid 3’-monooxygenase-like, flavonol synthase and isoflavone reductase homologs were upregulated, suggesting induced flavonoid production in infected plants. Various secondary metabolites have been shown to protect the plant by reducing the virulence and multicellular behavior of *D*. *solani* and Pectobacteria [55–58]. Vetispiradiene synthase, that according to KEGG is involved in the production of sesquiterpene phytoalexins, and a protein annotated as SGA, similar to rhamnose:beta-solanine/beta-chaconine rhamnosyltransferase (SGT3), and solanidine UDP-glucose glucosyltransferase 1 (SGT1) that is involved in glycosylation of the steroidal alkaloid aglycon solanidine [59], were downregulated. These results suggest reduced production of solanine in infected tuber tissue at the beginning of the infection. A similar reduction in glycoalkaloids was also evident in potato plants induced by *Phytophthora infestans* elicitors [60]. It seems that when the tubers allocate resources for phenylpropanoid and flavonoid production, they produce less toxic phytoalkaloids.

### Late response of symptomatic potato tuber to *Dickeya solani* inoculation

During the late, symptomatic phase of *D*. *solani* infection at the 168 hpi time point, 132 and 281 statistically significant DEGs were up- and downregulated, respectively (S3 Table). When compared to the 1 and 24 hpi data, the late response either contained different genes, or the same genes were regulated in the opposite direction. For example, apart from receptor-like CRK2, all other receptor-like kinases and other defense proteins that were upregulated at early time points were downregulated in the 168 hpi data, including WRKY70. According to GO enrichment analysis, the GO biological process cell wall organization was significantly enriched in the late response (Fig 4). Downregulated genes encoding xyloglucan transglucosylase/hydrolases, proline-rich proteins and cellulose synthases combined with upregulation of genes encoding extensin-like protein, expansin proteins and COBRA-like protein suggest changes in cell wall plasticity and integrity maintenance [61] in the infected potato tubers at late time points. In addition, genes involved in pectin degradation, such as pectinesterase, three pectate lyases and polygalacturonase, were upregulated, whereas 21 kDa protein-like, identified in the SwissProt database as pectin methylesterase inhibitor, was downregulated. In Arabidopsis, trimers and longer oligogalacturonides have been shown to lead to wound response and JA biosynthesis and signaling [11]. It appears that JA-dependent defense is activated during the necrotrophic phase of *D*. *solani* infection when a high bacterial population density produces PCWDEs to cause tissue maceration. The induced host pectinases may promote cell wall remodeling [62], which may increase the amount of defense-activating pectic fragments to amplify the DAMP response. On the other hand, plant pectinases and cell-wall modification have been suggested to be susceptibility responses that are induced by virulent plant pathogens to promote symptoms in their susceptible host plant [63].

RNA-Seq results revealed that genes and GO processes related to primary metabolism were altered in treated tubers at 168 hpi (Fig 4, S3 Table). In this category, genes involved in amino acid metabolism showed changes in gene expression, most of which were downregulated. Among the downregulated genes, three were identified as glutamate decarboxylase, which is involved in many biochemical pathways, including biosynthesis of the signaling molecule gamma-aminobutyric acid (GABA). The production of GABA protects plants against abiotic and biotic stresses but can be downregulated by virulent pathogens [64]. Genes involved in starch breakdown (e.g., plastid alpha amylases, apoplast invertase and bidirectional sugar transporter) were highly upregulated, suggesting increased sugar content in infected tubers, which might promote bacterial growth. Two upregulated hexokinases identified at the late time point may act as sugar sensors that regulate sugar-dependent gene repression or activation [65] and may regulate the execution of programmed cell death in plant cells [66]. Furthermore, 31 DEGs in GO term transport were differentially expressed at the 168 hpi time point, among them were several sugar transporters (S3 Table). The function of sugar transporters may be important for the plant’s ability to mount an efficient defense, or it may be related to the ability of the bacteria to modulate sugar efflux of their host for their own benefit [67]. Redistribution of sugars and changes in the transport of water and nutrients have been identified as susceptibility responses affected by pathogens to create a suitable environment for pathogenesis [63].

Arginine decarboxylase and S-adenosylmethionine decarboxylase, which are involved in the biosynthesis of polyamines, were upregulated, as were several polyamine oxidase transcripts involved in spermidine biosynthesis. Polyamine production has been linked to wound healing in potato tubers and resistance to *Pseudomonas* in Arabidopsis [68, 69]. Significant changes were observed in the expression levels of several genes involved in the biosynthesis of secondary metabolites. DEGs coding for squalene epoxidase (squalene monoxygenase), which is involved in sterol biosynthesis and has a vital role in membrane permeability, ROS regulation and secondary metabolite production, were upregulated, whereas six DEGs were identified by KEGG as cytochrome P450, most of which were downregulated (S3 Table). One of them functions most likely as fatty acid omega-hydroxylase, involved in suberin and cutin biosynthesis, four of the cytochrome P450s were annotated as premnaspirodiene oxygenase-like in the potato genome, and the two upregulated cytochrome P450s were identified in the KEGG database as being involved in the biosynthesis of solavetivone and rishitin, sesquiterpene phytoalexins of potato [70]. Rishitin is toxic to soft rot bacteria and could be a factor that protects the tubers from rotting [71].

Linoleic acid metabolism, fatty acid biosynthesis and biosynthesis of unsaturated fatty acids were among the top significantly enriched KEGG pathways during the symptomatic phase of tuber infection (Fig 4). Among them, 10 DEGs, mostly downregulated, were linked to fatty acid, phospholipid or sphingolipid metabolism, and most of the downregulated genes were present in the plasma membrane or the cell wall [72]. Two lipoxygenases were upregulated, and they were annotated as 5-lipoxygenase and linoleate 9S-lipoxygenase 6-like, which are involved in the production of oxylipins affecting plant cell death responses and pathogen defense [73, 74]. However, linoleate 13S-lipoxygenase 3 (LOX-H3), which is involved in the insect-induced wound response but not JA biosynthesis in potato [75], was downregulated.

### Expression of genes involved in biosynthesis and signaling of defense phytohormones

**Fig 5:**
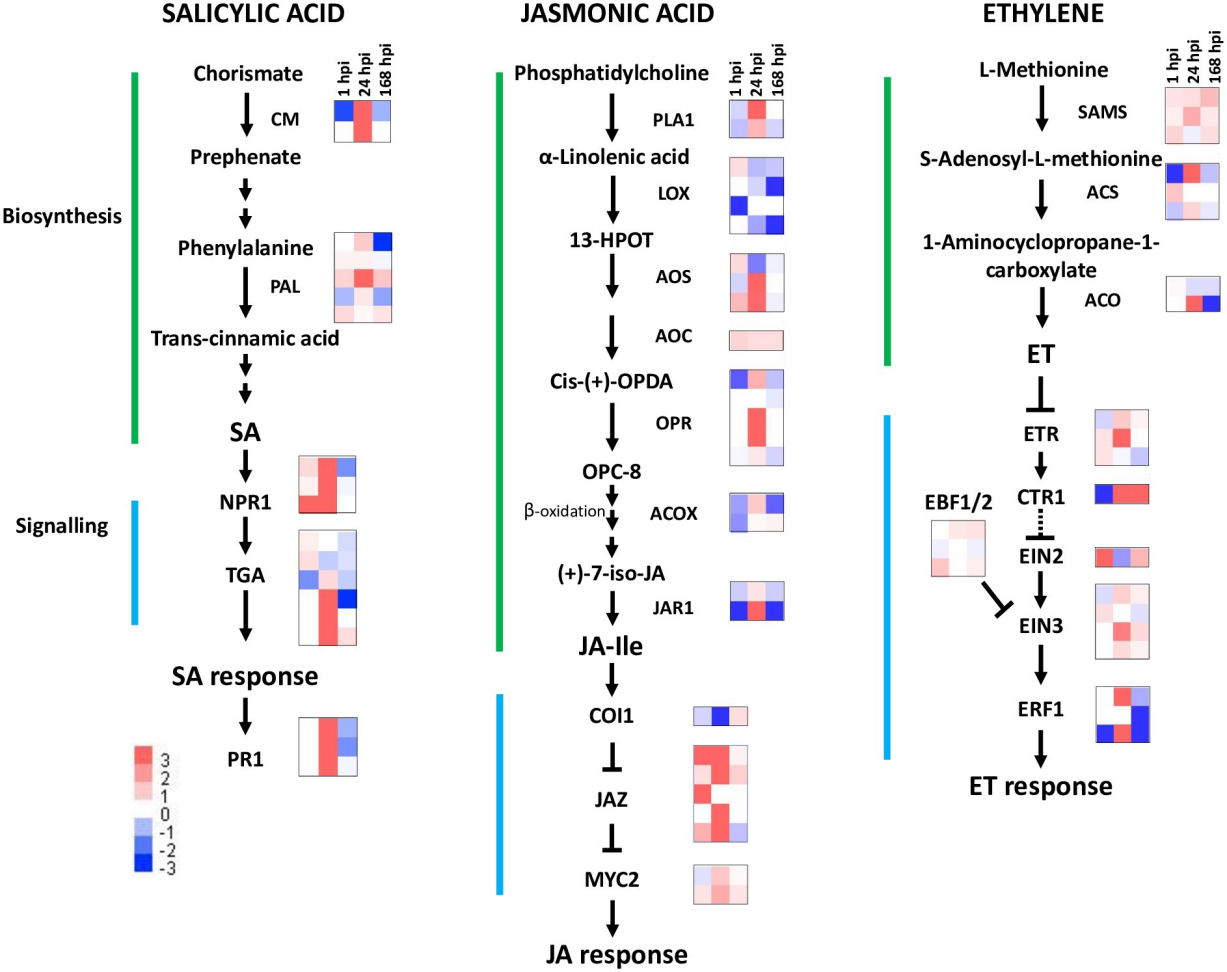
Pathways and heatmaps of RNA-Seq data showing DEGs linked to salicylic acid (SA), jasmonic acid (JA) and ethylene (ET). The SA biosynthesis pathway was adopted from Lefevere et al., 2020 [76], the JA biosynthesis pathway from Wasternack and Hause 2013 [83], and the ET pathway from Dubois et al., 2018 [84]. Hormonal signaling was adopted from the KEGG pathway “sot04075, plant hormone signal transduction” of potato. CM, chorismate mutase; PAL, phenylalanine ammonia-lyase; NPR1, regulatory protein NPR1; TGA, transcription factor TGA; PR1, pathogenesis-related protein 1; PLA1, phospholipase A1; LOX, lipoxygenase; AOS, allene oxide synthase; AOC, allene oxide cyclase; OPR, 12-oxophytodienoic acid reductase; ACOX, acyl-CoA oxidase; JAR1, jasmonic acid-amino synthetase; COI1, coronatine-insensitive protein 1; JAZ, jasmonate ZIM domain-containing protein; MYC2, transcription factor MYC2; SAMS, S-adenosylmethionine synthase; ACS, 1-aminocyclopropane-1-carboxylate synthase; ACO, aminocyclopropanecarboxylate oxidase; ETR, ethylene receptor; CTR1, serine/threonine-protein kinase CTR1; EIN2, ethylene-insensitive protein 2; EIN3, ethylene-insensitive protein 3; EIL, Ethylene insensitive 3-like; EBF, EIN3-binding F-box protein; ERF, ethylene-responsive transcription factor. The expression levels of individual genes are shown in S2 Table.

Many of the identified statistically significant DEGs were involved in the regulation of the SA, JA and ET signaling pathways, as evidenced by the KEGG term “plant hormone signal transduction” and the GO term “hormone-mediated signaling” at all time points (Fig 4). However, very few genes involved in the biosynthesis of the hormones were among the significant genes shown in S3 Table. Therefore, all DEGs that could be linked to SA, JA and ET signaling were identified in S2 Table, and those verified by Blast analysis in the KEGG database were used in heatmap analysis (S6 Table, Fig 5). The SA biosynthesis gene chorismate mutase (CM) was upregulated only at 24 hpi, but phenylalanine ammonia-lyase genes were observed at all time points. SA biosynthesis can take place by two pathways in plants: the isochorismate synthase (ICS) and phenylalanine pathways [76]. No ICS was identified among the potato transcripts, which indicates that the production of SA takes place via the phenylalanine route in potato tubers upon *D*. *solani* infection. This finding supports the conclusion that similar to tobacco [77, 78], the phenylalanine ammonia-lyase pathway is the main biosynthetic pathway of pathogen-induced SA in potato. Additionally, SA regulators NIM1-like protein 1, NIM1-like protein 2 and NPR5, all identified in the KEGG database as NPR1, and DNA-binding proteins TGA1, TGA7 and TGA2.1-like showed the clearest upregulation at the 24 hpi time point. SA-induced genes, basic form of pathogenesis-related protein 1A1 and pathogenesis-related protein 1-like as well as other PR proteins, and transcription factors that regulate downstream responses to salicylic acid (MYB48, WRKY3 and WRKY70) [38, 39, 79] were induced. In conclusion, the results suggested that SA biosynthesis and signaling were activated in the early samples. These results are in agreement with earlier studies suggesting that molecules produced by soft rot bacteria induce SA production, which is needed for the full defense of their host plant [reviewed recently in 4].

Most genes involved in JA biosynthesis showed the clearest upregulation at the 24 hpi time point; the only exceptions were the genes identified as 13S-lipoxygenase in KEGG analysis, for which only weak upregulation was observed at the 1 hpi time point (S6 Table, Fig 5). Differences between the SOL database and NCBI were detected, and two 13S-lipoxygenases listed in the KEGG database were not present in the SOL database. Thus, it is possible that some 13S-lipoxygenase genes were not identified in our analysis. Five DEGs encoding jasmonate ZIM-domain containing proteins (JAZ) negatively affecting JA signaling had the highest upregulation at the early time points. Jasmonate receptor coronatine insensitive protein 1 was upregulated only at the 168 hpi time point, while transcription factor MYC2 and MYC2-like transcripts were slightly upregulated at all time points. Furthermore, serine/threonine-protein kinase OXI1-like kinase, which is needed for JA production during high-light stress, ROS-induced cell death and resistance in *Pseudomonas tomato*-infected Arabidopsis [80, 81], was downregulated in the 1 hpi sample. Based on the expression of JA signaling and downstream genes, JA biosynthesis seemed to occur at early time points, while signaling was most effective at late time point. These results support an earlier conclusion that JA production is a typical response of the host plant during symptomatic phase of the infection caused by soft rot bacteria [4, 5, 82].

ET biosynthesis and signal transduction were affected in the infected potato tubers at all time points (S6 Table, Fig 5). The gene identified in the KEGG database as CTR1-like protein kinase, which acts as a negative regulator of the ethylene response, was downregulated only at 1 hpi, and a gene annotated as ethylene signaling protein, similar to EIN2 acting positively on ethylene-mediated gene regulation, was upregulated at 1 hpi, both changes suggesting an enhanced ethylene response at 1 hpi. DEGs encoding the ethylene response factors ERF1 and ERF1-like were upregulated only in the 24 hpi dataset. The ERF2-like gene, a regulator needed for JA signaling and pathogen defense in tomato [85], was upregulated at the late time point, possibly positively affecting JA production in potato tubers.

**Fig 6:**
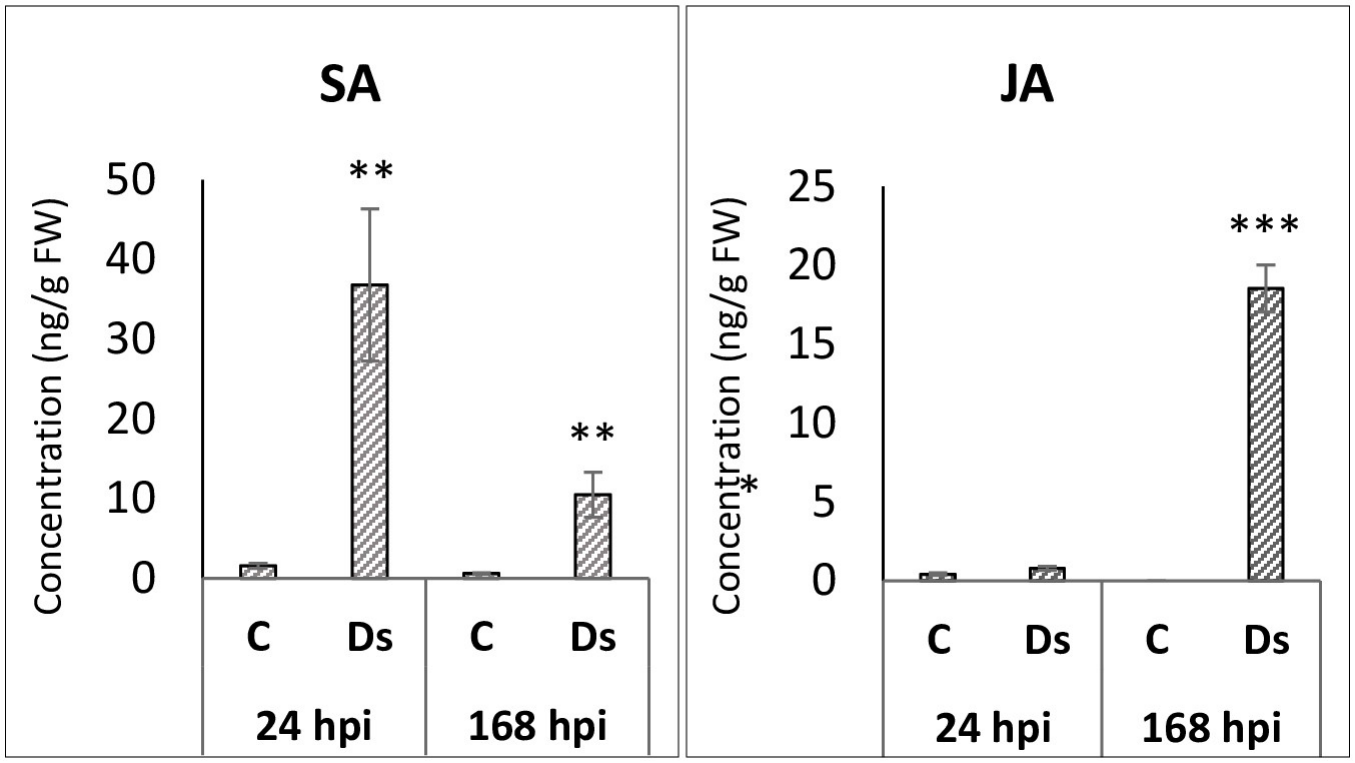
Salicylic acid (SA) and jasmonic acid (JA) concentrations in tubers inoculated with *Dickeya solani* and corresponding control tubers 24 h and 168 h post-inoculation (hpi). Changes in tuber phytohormone contents (ng/g fresh weight) in controls (C) and inoculated tubers (Ds). Stars indicate significant differences in the t-test.

Because SA and JA biosynthesis and signaling seemed to differ in the early and late samples, the concentrations of endogenous SA and JA were determined in *D*. *solani*-infected tubers at two time points, 24 and 168 hpi, using the LCMS/MS system. The results showed that the SA concentration increased in the infected tubers at 24 hpi and then decreased in the symptomatic tubers at 168 hpi (Fig 6). Almost no JA was produced at the beginning of the infection at 24 hpi in the asymptomatic tubers despite the induction of the biosynthesis genes at the transcriptional level. It is possible that the translation of the transcripts was prevented by post-transcriptional regulation, or that JA was modified to a form that was not identified by the LCMS/MS analysis. Increased JA concentration was measured in the symptomatic tubers showing rotting symptoms. The turning point from asymptomatic infection to active rotting may depend on the growth of the bacteria. When the *D*. *solani* concentration increases in the inoculation point and reaches the concentration that activates quorum sensing [86], the bacteria produce more pectic enzymes that increase the cell wall damage and production of pectic fragments, which upregulates JA biosynthesis and signaling [87].

Previously, induced JA concentration has been identified when phytohormone concentrations have been measured after inoculation with soft rot bacteria [88–90], and the transcriptome profiling has shown upregulation of JA biosynthesis or signaling genes in infected plants [82, 91], whereas increased SA biosynthesis has been previously measured only from *Dickeya dadantii*-inoculated resistant *N*. *benthamiana* plants [90], and from Arabidopsis leaves inoculated with *Pectobacterium versatile* [90]. SA production was reported to drop after initial slight induction in potato leaves treated with *P*. *parmentieri* [86], and the gene expression data from *in vitro* tobacco plants inoculated with *P*. *atrosepticum* also suggested downregulation of SA production [82]. It seems that the SA and JA concentrations may depend on the susceptibility of the host, the pathogen species and its virulence, the incubation conditions and the time of sampling.

## Conclusions

The goal of this work was to study how potato tubers respond to inoculation by *D*. *solani* in asymptomatic and symptomatic tubers. The results show that even the artificially inoculated tubers that are fully susceptible and are incubated in conditions that eventually lead to rotting, they react with the PTI-like response, defense gene expression and SA production in the early phase of the infection. Additionally, ET and possibly also low level of JA or other jasmonates are produced in the beginning of the infection, leading to wall-associated defenses, induction of proteinase inhibitors and production of flavonoid and phenylpropanoid secondary metabolites, but downregulated production of steroidal glycoalkaloid solanine. In the late phase of infection in symptomatic tubers, SA concentration is reduced, and JA concentration is increased, which leads to intensified cell wall-related responses and increased production of the antibacterial sesquiterpene phytoalexin rishitin. In the symptomatic tubers, genes that have been described as susceptibility factors are also activated, including pectic enzymes. Even when the tubers are susceptible and show signs of rotting, the JA response most likely slows down the infection and reduces rotting, as evidenced by previous results showing that mutations affecting either SA or JA biosynthesis increase rotting of potato stems and tubers when inoculated by *D*. *solani* [92]. Our results are consistent with previous studies suggesting that *D*. *solani* may act as a hemibiotrophic pathogen with a biotrophic phase followed by a switch to a necrotrophic phase in the later stage of the infection [21].

## Supporting information

S1 Fig qRT–PCR validation of DEGs obtained from RNA-Seq sequencing of potato tubers inoculated with *D*. *solani*. Quantitative measurement of gene expression was determined with qRT–PCR for 9 DEGs at 1 hours post inoculation (hpi), 11 DEGs at 24 hpi and 10 DEGs at 168 hpi. Data were obtained from three independent cDNA sets from three independent experiments, normalized to eukaryotic elongation factor 5A3 and expressed as the means of log2 (ΔΔCt) ± SEM (standard error of the mean). The graphs demonstrate the means of the log2 fold changes from three independent experiments. Error bars show the standard error of the mean, and statistical tests were performed with t-tests. Primers and full names of the genes are shown in S4 Table.
(TIF)

S1 Table Primary RNA-Seq data. (XLSX)

S2 Table All identified transcripts. Transcripts at 1, 24 and 168 hours post-inoculation in RNA-Seq data of potato tubers inoculated with *Dickeya solani* identified with NOISeq.
(XLSX)

S3 Table Statistically significant differentially expressed genes at 1, 24 and 168 hours post-inoculation. RNA-Seq data of potato tubers inoculated with *Dickeya solani* with probability > 0.75, p-value < 0.05 and log2 fold change ≥ 9 and ≤ −9.
(XLSX)

S4 Table Primers used in this study. (DOCX)

S5 Table Primary qRT–PCR data. (XLSX)

S6 Table Identity of the genes used for heatmap analysis to visualize SA, JA and ET pathways in inoculated tubers. (XLSX)
