# Supplementary figures and images for: Gene expression and phytohormone levels in the asymptomatic and symptomatic phases of infection in potato tubers inoculated with Dickeya solani
Source: PLoS One. 2022 Aug 29;17(8):e0273481. doi: 10.1371/journal.pone.0273481 (PMC9423618; doi:10.1371/journal.pone.0273481)

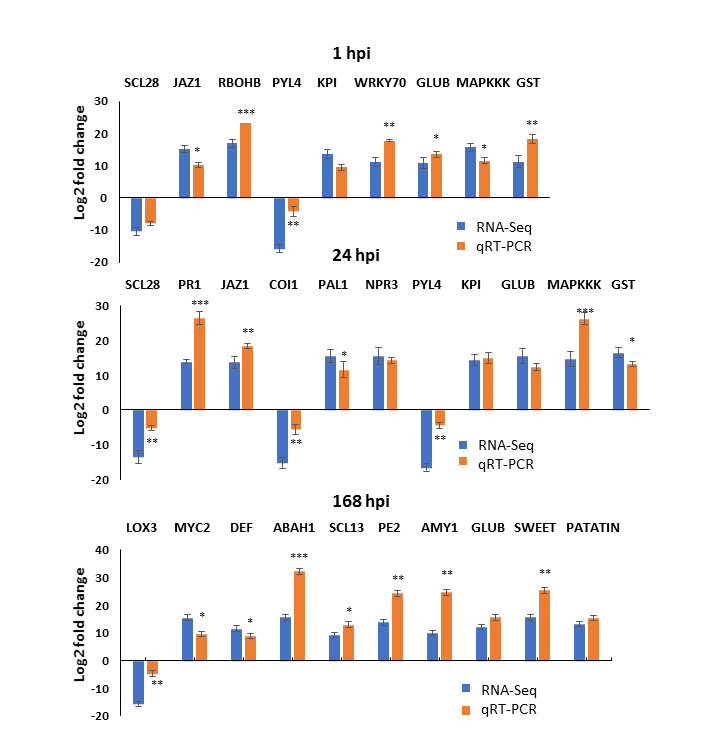

Supplement: S1 Fig — Quantitative measurement of gene expression was determined with qRT–PCR for 9 DEGs at 1 hours post inoculation (hpi), 11 DEGs at 24 hpi and 10 DEGs at 168 hpi. Data were obtained from three independent cDNA sets from three independent experiments, normalized to eukaryotic elongation factor 5A3 and expressed as the means of log2 (ΔΔCt) ± SEM (standard error of the mean). The graphs demonstrate the means of the log2 fold changes from three independent experiments. Error bars show the standard error of the mean, and statistical tests were performed with t-tests. Primers and full names of the genes are shown in S4 Table. (TIF) [file pone.0273481.s001.tif]
