# Supplementary material for: Gene expression and phytohormone levels in the asymptomatic and symptomatic phases of infection in potato tubers inoculated with Dickeya solani
Source: PLoS One. 2022 Aug 29;17(8):e0273481. doi: 10.1371/journal.pone.0273481 (PMC9423618; doi:10.1371/journal.pone.0273481)
Supplement: S4 Table — (DOCX) [file pone.0273481.s005.docx]

**Supplementary file S4** Primers used in this study.

| **Annotation** | **Gene/**  **Abbreviation** | **Primer sequences** |
| --- | --- | --- |
| Scarecrow-like protein 28 | SCL28 | RLG2-F: GCTCCTCTGTCCACAGGTCTTA  RLG2-R: TTTCCCTACGACTTGCATGTGC |
| PR1 protein precursor | PR1 | PR1-F: GTGCTGTGAAGATGTGGGTAGATG  PR1-R: CTATACTCAGGTAGTGTGGCGTAACT |
| TIFY 10A-like jasmonate repressor (JAZ1) | JAZ1 | JAZ1-F: TGAGAAATCTGGTGAGTCGGTTCAG  JAZ1-R: GGTTCCGATTCAGCCTTCATTGC |
| Coronatine-insensitive 1 | COI1 | COI1-F: GTGGCAGATCGATGCGATTACT  COI1-R: GAAAGGGAAACCAAGAGCTGCT |
| Phenylalanine ammonia-lyase 1 | PAL1 | PAL1-F: GTCAGATTGAGGCTGCTGCTATT  PAL1-R: CAAGATCGCTATGCTCTCCGAAC |
| NPR3 | NPR3 | NPR3-F: AGATGGAGCCTACGCTCTTCATTA  Npr3-R: CATTAGCAACACCCAGTCCAAGAA |
| Respiratory burst oxidase homolog protein B | RBOHB | RBOHB-F: GGTGTTGTGATGGTGGTCCTTATG  RBOHB-R: GTTGATGGAAGATTGAGTCGGTTTCT |
| Abscisic acid receptor PYL4 | PYL4 | PYL4-F: TGTTCGACGGAAGTACCCGATTCT  PYL4-R: GGTGGAGACGGGAGCTGAGAT |
| Kunitz-type enzyme inhibitor | KPI | KPI-F: CAAGACGGCGATCCAGTCAAGATT  KPI-R: CAGCAACACATCGTTTGGGCATTT |
| WRKY transcription factor 70 | WRKY70 | WRKY70-F: GAGGAGGAAGAATCCAAAGGACAAAG  WRKY70-R: AGCAGAGGGAGAAGAAGGCATAA |
| Glucan endo beta-1,3-glucanase | GLUB | GLUTP1-F: ATGCGAGATGGTGGGTACAGA  GLUTP1-R: GTGCCAGTAACAGGGCTGATTT |
| Mitogen-activated protein kinase kinase kinase NPK1-like | MAPKKK | MAPKKK-F: TTGGGCTGGCAAAGAGAATTGA  MAPKKK-R: GACCATTTCCGGCGACATGTATAG |
| Glutathione S-transferase | GST | GST-F: TGCTCCCTTCTGACCCTTATGT  GST-R: GTTCGTCTCCCGTTGCTGAATAAA |
| Lipoxygenase 3-1 | LOX3 | LOX-F: TTGCTTTACTCCTGGTCGCTACTG  LOX-R: GTTTCAGCCCATGAGGTTGTGTTG |
| Transcription factor MYC2 | MYC2 | MYC2-F: CAGCACAATAGAGCGAGCAACC  MYC2-R: TCTAAGGAGCGTTGTCCCTAACG |
| Defensin J1-2 | DEF | DEF-F: AGCCTCTTCACTCTTCTCCCTTC  DEF-R: ACTCCCTCCGATAGGAAAGCTTAAT |
| ABA 8'-hydroxylase 1 | ABAH1 | ABAH1-F: CGATGCTCGTTGTCCTGATGTT  ABAH1-R: GAGGCATCACATGAGCTTGACA |
| Scarecrow-like protein 21 | SCL21 | SCL21-F: GCACGGGATGTTGTCAATCTCATAG  SCL21-R: GATTTACACCATGCCCACTGAGTC |
| Pectinesterase 2 | PE2 | PE2-F: GCCGAACTGACCCGAATCAAA  PE2-R: TTTGCCACGGCCTTCCTAAAT |
| Alpha-amylase | AMY1 | AMY1-F: CAGTCCTTCAGGAAGCAGTCAAAG  AMY1-R: CCCTTGAAGGCCACAATCCTAAA |
| Sugar transporter protein 13 | STP13 | STP13-F: CCGGAGGACTCATGTTTGGTTATG  STP13-R: AACCTGGCTCCTTTGTTCTCTTG |
| Bidirectional sugar transporter SWEET12 | SWEET | SWEET-F: AAGCCAGGGTCCAAACAGTAAAG  SWEET-R: GTCCACGAATGGCTCCTTTGAA |
| Patatin storage protein | PATATIN | PATATIN-F: CGTTGGGAGAAATGGTGACTGTTC  PATATIN-R: TGTCCACATCCTGAAGTTGTCCT |
